# Accessory Factors of Cytoplasmic Viral RNA Sensors Required for Antiviral Innate Immune Response

**DOI:** 10.3389/fimmu.2016.00200

**Published:** 2016-05-25

**Authors:** Hiroyuki Oshiumi, Takahisa Kouwaki, Tsukasa Seya

**Affiliations:** ^1^Department of Immunology, Graduate School of Medical Sciences, Kumamoto University, Kumamoto, Japan; ^2^Precursory Research for Embryonic Science and Technology (PRESTO), Japan Science and Technology Agency (JST), Kumamoto, Japan; ^3^Department of Microbiology and Immunology, Graduate School of Medicine, Hokkaido University, Sapporo, Hokkaido, Japan

**Keywords:** type I IFN, RIG-I-like receptors, innate immune response, viruses, signaling pathway

## Abstract

Type I interferon (IFN) induces many antiviral factors in host cells. RIG-I-like receptors (RLRs) are cytoplasmic viral RNA sensors that trigger the signal to induce the innate immune response that includes type I IFN production. RIG-I and MDA5 are RLRs that form nucleoprotein filaments along viral double-stranded RNA, resulting in the activation of MAVS adaptor molecule. The MAVS protein forms a prion-like aggregation structure, leading to type I IFN production. RIG-I and MDA5 undergo post-translational modification. TRIM25 and Riplet ubiquitin ligases deliver a K63-linked polyubiquitin moiety to the RIG-I N-terminal caspase activation and recruitment domains (CARDs) and C-terminal region; the polyubiquitin chain then stabilizes the two-CARD tetramer structure required for MAVS assembly. MDA5 activation is regulated by phosphorylation. RIOK3 is a protein kinase that phosphorylates the MDA5 protein in a steady state, and PP1α/γ dephosphorylate this protein, resulting in its activation. RIG-I and MDA5 require cytoplasmic RNA helicases for their efficient activation. LGP2, another RLR, is an RNA helicase involved in RLR signaling. This protein does not possess N-terminal CARDs and, thus, cannot trigger downstream signaling by itself. Recent studies have revealed that this protein modulates MDA5 filament formation, resulting in enhanced type I IFN production. Several other cytoplasmic RNA helicases are involved in RLR signaling. DDX3, DHX29, DHX36, and DDX60 RNA helicases have been reported to be involved in RLR-mediated type I IFN production after viral infection. However, the underlying mechanism is largely unknown. Future studies are required to reveal the role of RNA helicases in the RLR signaling pathway.

## Introduction

The innate immune system is the first line of defense against viral infection. RIG-I-like receptors (RLRs) are cytoplasmic viral RNA sensors that recognizes viral double-stranded (ds) RNA and trigger antiviral innate immune responses (1). The RIG-I and MDA5 proteins, which are members of RLRs, comprise two caspase activation and recruitment domains (CARDs), a helicase domain, and a C-terminal domain (CTD) (2). The helicase domain and CTD bind to viral RNA, and CTD is essential for the recognition of viral RNA (3, 4). After the recognition of viral RNA, two N-terminal CARDs forms a two-CARD tetramer structure, which acts as a core of the MAVS prion-like aggregation structure (5, 6). The MAVS protein is a solo adaptor of RLRs and activates kinases and ubiquitin ligases, leading to the activation of transcription factors, such as IRF-3 and NF-κB (7–10). The transcription factors induce type I interferon (IFN) and pro-inflammatory cytokine production.

RIG-I recognizes relatively short dsRNA (<1 kbp) with a 5′ tri- or di-phosphate group (11–13), whereas another RLR member, MDA5, recognizes long dsRNA (>1 kbp) with or without a 5′ phosphate group (12). Influenza A virus, Sendaivirus, hepatitis C virus (HCV), and vesicular stomatitis virus (VSV) are mainly recognized by RIG-I, whereas encephalomyocarditis virus (EMCV) and poliovirus are recognized by MDA5 (14, 15). West Nile virus and Japanese encephalitis virus are recognized by both RIG-I and MDA5 (14, 15).

## Post-Translational Modification of RLRs

**Figure 1:**
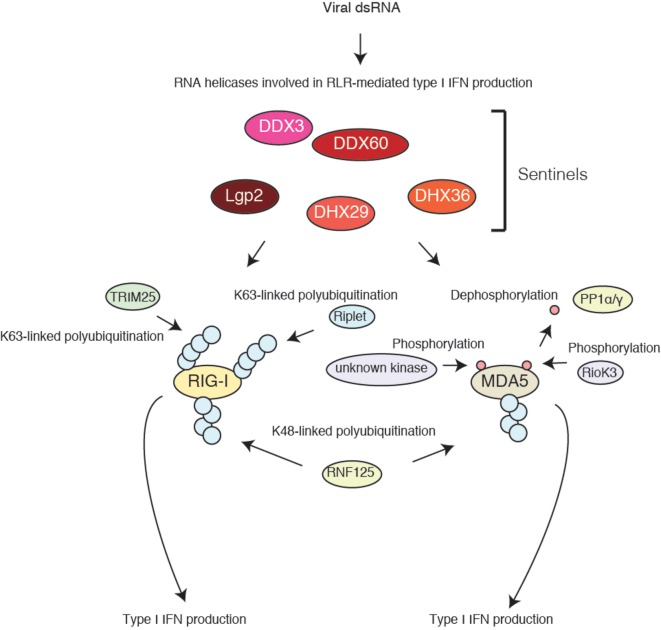
**Accessory factors of RIG-I and MDA5**. RIG-I and MDA5 undergo post-translational modifications. TRIM25 and Riplet ubiquitin ligases deliver a K63-linked polyubiquitin moiety to RIG-I N-terminal CARDs and C-terminal regions, respectively, resulting in type I IFN production. MDA5 is phosphorylated in resting cells. RIOK3 is a protein kinase involved in MDA5 phosphorylation; however, there seem to be other kinases targeting MDA5. PP1α/γ dephosphorylates MDA5, leading to type I IFN production. Several cytoplasmic RNA helicases are involved in RLR-mediated type I IFN production, but their biochemical activities are largely unknown.

Recent studies have revealed the post-translational modification of RLRs (Figure 1). Gack and colleagues first reported the K63-linked polyubiquitination of RIG-I CARDs by TRIM25 ubiquitin ligase, which is essential for their activation (16). Later studies showed that the non-covalent binding of the K63-linked polyubiquitin chain is sufficient to activate RIG-I signaling (17). A covalent and/or non-covalent K63-linked polyubiquitin chain stabilizes the two-CARD tetramer structure (6). Another ubiquitin ligase, Riplet (also called RNF135 or REUL), mediates K63-linked polyubiquitination of RIG-I C-terminal region, which promotes the binding of TRIM25 to RIG-I (18, 19). The knockout (KO) of each ubiquitin ligase has been shown to markedly reduce RIG-I-mediated type I IFN production (16, 20). These ubiquitin ligases are targeted by several viral proteins, such as NS-1 of influenza A virus and NS3-4A of HCV, resulting in the attenuation of RIG-I-mediated type I IFN production (19, 21). These findings indicate the importance of both ubiquitin ligases in RIG-I activation. RIG-I also undergoes K48-linked polyubiquitination by RNF125, leading to its proteasomal degradation (22). TRIM25 itself undergoes linear polyubiquitination by the linear ubiquitin assembly complex (23). These observations indicate that the ubiquitin chain plays a critical role in the cytoplasmic antiviral innate immune response (24).

MDA5 assembles along viral long dsRNA and forms a nucleoprotein filament structure required for MAVS activation (25). MDA5 activation is regulated by phosphorylation (26). In resting cells, the MDA5 protein undergoes phosphorylation. PP1 α/γ are required for MDA5 dephosphorylation, leading to its activation. Recently, we reported that RIOK3 is a protein kinase that mediates MDA5 phosphorylation at Ser-828, which impairs MDA5 assembly and attenuates its activation (27). Considering that Gack and colleagues have shown that the N-terminal region of MDA5 is phosphorylated (26), other protein kinases that phosphorylate MDA5 are required for the attenuation of the signaling.

The phosphorylation of RIG-I and ubiquitination of MDA5 have also been reported and are required for the regulation of RLR signaling (24).

## RNA Helicases Involved in RLRs-Mediated Type I IFN Production Pathway

Several RNA helicases lacking CARDs are involved in RLR-mediated signaling (28). Bowie and colleagues first reported that a non-RLR helicase, DDX3, is involved in RIG-I-mediated type I IFN production. They showed that DDX3 is required for the activation of TBK1 and IKK-ɛ, which are downstream factors of MAVS (29, 30). Later, we reported that DDX3 associates with RIG-I and promotes RIG-I–RNA binding (31).

LGP2 is a member of RLRs but lacks N-terminal CARDs (2). Therefore, LGP2 by itself cannot trigger the signal to induce type I IFN. Initial studies reported that LGP2 attenuates RLR signaling in response to polyI:C in mouse embryonic fibroblasts (MEFs) (2, 32), whereas later studies reported a positive role of LGP2 in RLR signaling during viral infection (33). DHX36 is a cytoplasmic RNA helicase and does not comprise CARDs. DHX36 protein complexed with DDX1 and DDX21 recognizes viral RNA and triggers the signal to induce type I IFN production via TRIF/TICAM-1 adaptor in some kinds of dendritic cells (DCs) (34). DHX36 also functions upstream of RIG-I and is required for the formation of antiviral stress granules where viral RNA is recognized by RLRs (35). DHX29 RNA helicase directly binds to polyI:C and polydA:dT and also associates with RIG-I. The protein exhibits a cell-type-specific expression pattern and is required for type I IFN production only in human respiratory epithelial cells (36).

DDX60 is another cytoplasmic RNA helicase, which does not contain N-terminal CARDs as RNA helicases described above. The DDX60 protein binds to dsRNA and associates with RLRs (37). DDX60 promotes RIG-I–RNA binding, which triggers type I IFN production (37). Previously, we generated *DDX60* KO mice and reported that *DDX60* KO moderately reduced RIG-I-mediated type I IFN production from peritoneal macrophages and MEFs but not from bone-marrow-derived cells, suggesting the cell-type specificity (38). DDX60 exhibited antiviral activities against only specific viruses (39). Another group also generated a DDX60 gene trapping mouse (called *DDX60* KO first) and a *DDX60* KO mouse (called *DDX60* KO full) (40), whose constructs are different from our *DDX60* KO mouse construct (38, 40). They confirmed that bone-marrow-derived cells of *DDX60* KO first and KO full mice normally produced type I IFN as we observed in our *DDX60* KO mice (40). Although *DDX60* KO first seems to reduce type I IFN production after stimulation with a low concentration of a RIG-I ligand, type I IFN production from *DDX60* KO first MEFs were comparable to wild-type MEFs in other experimental conditions (40). These data implied that DDX60 is not a general factor for RIG-I activation and plays a role in RIG-I signaling only when cells are infected with specific viruses or stimulated with specific or low concentration of RIG-I ligands in a cell-type-specific manner (38–40).

Recently, we identified another role of DDX60 in the antiviral response. The DDX60 protein exhibits the similarity to SKI2 RNA helicase, a component of the RNA exosome, which degrades host and viral RNA (37, 40). We found that DDX60 associates with the core components of the RNA exosome and is involved in a viral RNA degradation pathway (37, 38). DDX60-mediated viral RNA degradation plays an important role in the antiviral response when the RIG-I pathway is blocked (38).

## Perspective

**Figure 2:**
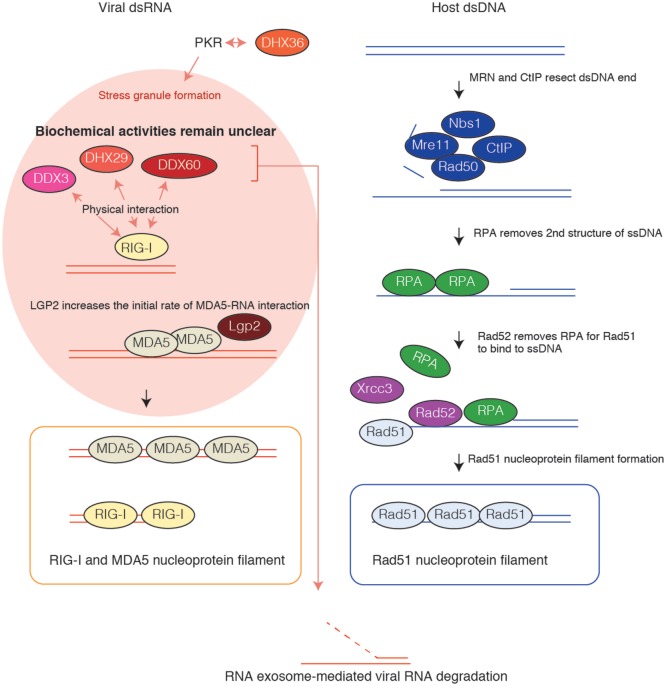
**Nucleoprotein filament formation**. Rad51 assembles along the single-stranded (ss) DNA region and forms a nucleoprotein filament. Mre11/Rad50/Nbs1 protein complex resects DNA double-stranded (ds) DNA together with CtIP, leading to the production of the ssDNA region. First, RPA binds to the ssDNA region to prevent the formation of secondary structure. Next, Rad52 and other proteins remove RPA from DNA for Rad51 to assemble along the ssDNA. RIG-I and MDA5 assemble along viral dsRNA and form nucleoprotein filaments. LGP2 modulates MDA5 nucleoprotein filament formation, resulting in type I IFN production. DHX36 is required for PKR-mediated antiviral stress granule formation. DDX3, DHX27, and DDX60 bind to RIG-I. The biochemical activities of DDX3, DHX29, DHX36, and DDX60 RNA helicases in nucleoprotein filament formation are largely unknown.

RIG-I and MDA5 forms a nucleoprotein filament (41). It is well known that the Rad51 protein, which is involved in DNA homologous recombination, forms a nucleoprotein filament along single-stranded (ss) DNA (42–45). For nucleoprotein filament formation, Rad51 requires several accessory factors. The Mre11 protein complex produces an ssDNA region (46), and then the RPA and Rad52 cooperatively produce the platform for Rad51 nucleoprotein filament formation as described in Figure 2 (47–49). There are several other factors involved in the Rad51 pathway, which occasionally compensates for a defect of other factors (50, 51). The role of each factor has been revealed by intensive biochemical studies. By contrast, the biochemical activities of accessory factors for RIG-I and MDA5 are largely unknown (Figure 2). Recently, Horvath and colleagues have revealed that LGP2 regulates MDA5 filament assembly (52).

The LGP2 protein increases the initial rate of MDA5–RNA interaction, resulting in the formation of numerous shorter MDA5 filaments (52). These numerous shorter filaments augment the signaling activity compared with that when there are fewer long MDA5 filaments. This supports the previous conclusion that LGP2 is not a negative factor but a positive factor for MDA5 signaling (33). Other accessory factors, such as DDX3, DHX29, DHX36, and DDX60, are expected to be involved in nucleoprotein filament formation. Biochemical analysis is required to clarify the role of accessory factors in RIG-I signaling.

## Author Contributions

All authors listed have made substantial, direct, and intellectual contribution to the work and approved it for publication.

## Conflict of Interest Statement

The authors declare that the research was conducted in the absence of any commercial or financial relationships that could be construed as a potential conflict of interest.
